# Impact of silk hydrogel secondary structure on hydrogel formation, silk leaching and in vitro response

**DOI:** 10.1038/s41598-022-07437-4

**Published:** 2022-03-08

**Authors:** Gemma Egan, Suttinee Phuagkhaopong, Saphia A. L. Matthew, Patricia Connolly, F. Philipp Seib

**Affiliations:** 1 Department of Biomedical Engineering, Faculty of Engineering, University of Strathclyde, Glasgow, UK; 2 Strathclyde Institute of Pharmacy and Biomedical Sciences, University of Strathclyde, 161 Cathedral Street, Glasgow, G4 0RE UK; 3 EPSRC Future Manufacturing Research Hub for Continuous Manufacturing and Advanced Crystallisation (CMAC), University of Strathclyde, Technology and Innovation Centre, 99 George Street, Glasgow, G1 1RD UK

**Keywords:** Biomedical engineering, Biomaterials - proteins, Biomedical materials

## Abstract

Silk can be processed into a broad spectrum of material formats and is explored for a wide range of medical applications, including hydrogels for wound care. The current paradigm is that solution-stable silk fibroin in the hydrogels is responsible for their therapeutic response in wound healing. Here, we generated physically cross-linked silk fibroin hydrogels with tuned secondary structure and examined their ability to influence their biological response by leaching silk fibroin. Significantly more silk fibroin leached from hydrogels with an amorphous silk fibroin structure than with a beta sheet-rich silk fibroin structure, although all hydrogels leached silk fibroin. The leached silk was biologically active, as it induced vitro chemokinesis and faster scratch assay wound healing by activating receptor tyrosine kinases. Overall, these effects are desirable for wound management and show the promise of silk fibroin and hydrogel leaching in the wider healthcare setting.

## Introduction

The current treatment strategies for chronic wounds typically include application of standard and advanced wound dressings^1^ and compression bandaging^2^. Ultrasound^3^, debridement^4^ and skin substitutes^5,6^ are more advanced therapeutic interventions, but the treatment of chronic wounds remains challenging and still requires orthogonal treatment strategies. Examples include physical methods, such as negative pressure wound therapy^7^ and real-time sensing applications that can support clinical decision making (e.g. when to change dressings^8^) and enable the early detection of infection^9^ (e.g. WoundSense, Ohmedics Ltd). Electrical stimulation to accelerate healing by reducing infection and increasing tissue perfusion is also promising^10–12^. A number of new products are now being approved for use in humans (e.g. WoundEL, PosiFect RD, Procellera).

Despite these advances, wound dressings continue to be the key staple of wound management; therefore, a broad spectrum of both synthetic and natural materials are now utilized (reviewed in^10,13^). One of the natural materials emerging as a promising biomaterial for wound care is silk. The silk fibroin protein is a clinically approved biomaterial that is widely used for load-bearing applications (e.g. sutures, surgical meshes)^14^. In 2019, the first reconstituted silk fibroin injectable (Silk Voice^®^, Sofregen Inc, Medford, MA, USA) gained clinical approval for vocal fold augmentation. The Silk Voice^®^ product has demonstrated that reconstituted silk fibroin can be acceptable for registration in the medical regulatory frameworks (e.g. The United States Food and Drug Administration). A small ongoing clinical trial (NCT04085822) sponsored by Silk Medical Aesthetics Inc (Medford, MA, USA) is examining the use of this technology to improve aesthetic and the results are eagerly awaited. Small-scale clinical trials using fibroin formulated as silk films^15^, sponges^16^ and knitted scaffolds^17,18^ have also shown favourable outcomes for both wound repair and aesthetics. This work is now being complemented by preclinical studies. For example, topical application of self-assembling silk hydrogel in a rabbit ear hyperplastic scar model showed significant therapeutic efficacy^19^. Liquid silk is currently used in cosmetics for topical application to the skin (e.g. Silk Therapeutics Inc).

Silk fibroin hydrogels that can self-assemble in vitro are capable of supporting comparable human fibroblast proliferation and keratinocyte migration to that obtained with collagen hydrogels^20^. In vivo, these silk hydrogels can serve as a support matrix for healing third-degree burn wounds in mice by guiding the local tissue response from a wound-mediated inflammatory response to the proliferative stage by orchestrating cell recruitment, cytokine signalling and extracellular matrix deposition^20^. Second-generation silk hydrogels have also shown improvements in wound healing in rodent models^21,22^, while silk hydrogels loaded with fibroblast growth factor 1^23,24^ and mesenchymal stem cells^25^ improved the healing rate, function and aesthetic appearance in vivo over their respective controls.

Silk is clearly making inroads into wound care (reviewed in^13^), and the first silk products are now appearing in the clinic arsenal^14^. The current paradigm is that the successful therapeutic response of these products is due to their content of solution-stable silk. However, this paradigm is now questioned by the finding that local application of soluble silk as a treatment for dry eye increased tear production, improved the smoothness of the cornea and reduced corneal epithelial detachment and inflammation^26^. Treatment of injured rabbit corneas with soluble silk accelerated the acute corneal epithelial healing process, resulting in the recovery of a robust multi-layered epithelium with increased tight function and focal adhesions^27^ (reviewed in^28^). The soluble silk is expected to adopt a random coil/alpha-helical secondary structure.

Therefore, emerging evidence suggests that soluble silk also has a significant impact on wound healing, and raises the possibility that soluble silk leaching from hydrogels may also play an important role.

No studies have yet assessed whether soluble silk can leach from silk fibroin hydrogels. One aim of this study was therefore to produce physically crosslinked silk hydrogels with tuned crystallinity to investigate the impact of the silk secondary structure on silk fibroin leaching. A second aim was to determine the biological response to this leached silk. The silk hydrogel secondary structure was tuned using electro-gelation and sonication. The silk fibroin content of the hydrogels was measured and the efficiency of the solution–gel transition was quantified. The biological response to leached silk was measured through fibroblast response by determining cell proliferation, migration and downstream molecular signalling.

## Results

**Figure 1:**
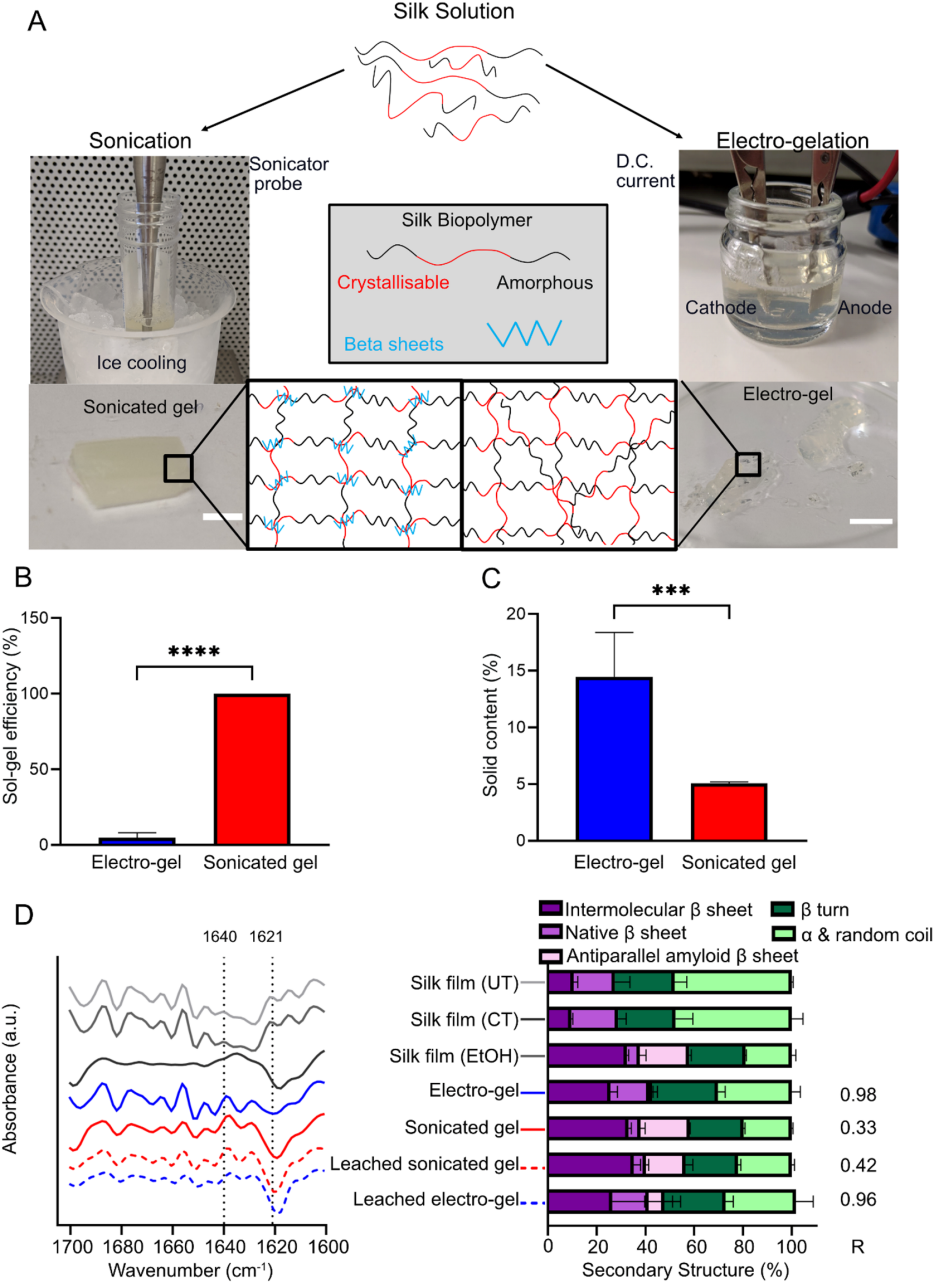
Production and characterisation of silk hydrogels. (**A**) Illustrative overview of the secondary structures found in silk solution and the changes seen with application of either ultrasonic waves or direct current, resulting in a sonicated hydrogel or an electro-gel, respectively (scale bar 0.25 cm). (**B**) Sol–gel efficiency of sonication or electro-gelation of silk fibroin solution (n = 5). (**C**) Solid content of the resultant electro-gel and sonicated gel (n = 5). (**D**) FTIR absorbance spectra of the amide I region of electro-gels and sonicated gels. Controls included were untreated air-dried silk film (UT) and the current treated solution remaining after removal of the electro-gel; this solution was air-dried into a film (CT). The third control was an ethanol treated silk film (EtOH) (n = 3). Leached samples in water of both electro-gel and sonicated gel after 72 h are included here. R is the correlation coefficient to freeze dried silk I. Lines at 1640 and 1621 indicate the amorphous and crystalline region, respectively (n = 8).

Physically cross-linked silk hydrogels were generated using sonication energy and electro-gelation (Fig. 1a). Gravimetric analysis of the silk content of both hydrogel types indicated significant differences (Fig. 1c). The sonicated hydrogel had a 100% solution–gel conversion efficiency, whereas the electro-hydrogel had a significantly lower efficiency (4.77 ± 3.24%) (Fig. 1b). The sonicated hydrogel showed a lower solid silk content (5% w/v) and less release, whilst the electro-hydrogels contained more silk (14.46 ± 3.91% w/v) but also released significantly more silk.

**Figure 2:**
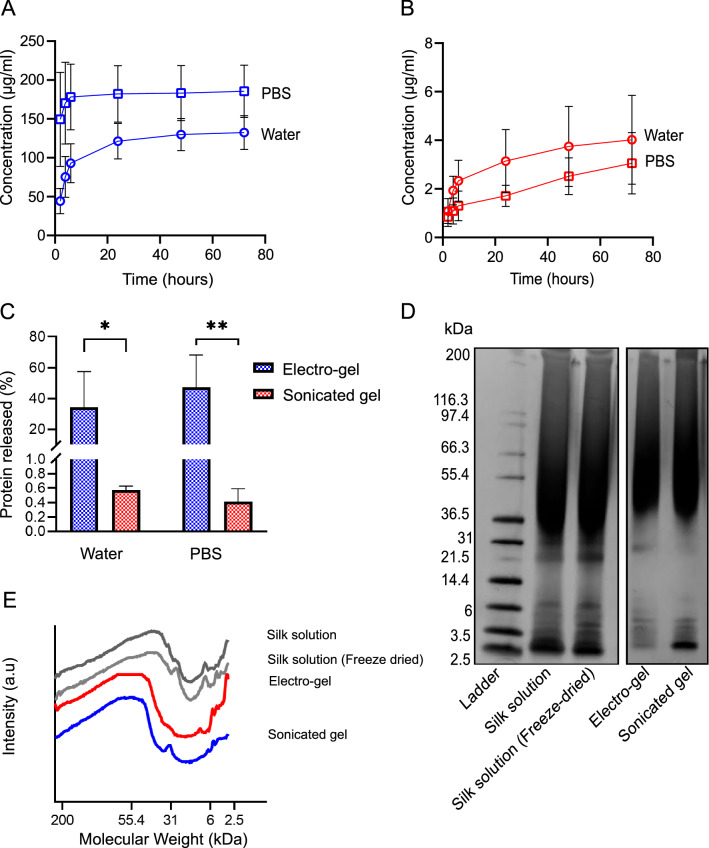
Protein release and characterisation from silk hydrogels measured by protein quantification and gel electrophoresis. (**A**) Electro-gel protein release in water and PBS over 72 h. (**B**) Sonicated hydrogel protein release in water and PBS over 72 h. (**C**) Total protein released after 72 h in water and PBS as a percentage of the starting quantity for electro-gels and sonicated gels (n = 4). (**D**) SDS PAGE of protein released from electro-gels and sonicated gels. Standards included are silk solution and freeze-dried silk solution reconstituted in water. (**E**) Densitometry analysis of SDS PAGE.

The ability of the gel to retain the incorporated silk was also examined and quantified over 72 h (Fig. 2a–c). For sonicated hydrogels, the percentage release from the starting concentration was 0.57 ± 0.05% in water and 0.41 ± 0.18% in PBS, with no significant difference between the release media. The pattern of release followed a zero order. By contrast, the electro-hydrogel showed a first order release profile, with most of the release occurring within the initial 12 h. The average cumulative release values at 72 h at 37 °C were 34.19 ± 23.27% and 47.40 ± 20.83% in water and PBS, respectively.

The secondary structure of the silk fibroin was measured by FTIR (Fig. 1d). The deconvoluted data indicated that the sonicated hydrogel was dominated by intermolecular beta sheets (33.14 ± 1.07%), with beta turns and antiparallel amyloid beta sheets being the next most common secondary configuration (21.98 ± 0.61% and 20.28 ± 0.05%, respectively). This contrasts with the electro-hydrogels, which displayed a different, statistically significant, distribution of secondary structures within the amide I region. Alpha and random coil structures were the most abundant (30.22 ± 3.55%), followed by beta turns and intermolecular beta sheets (27.11 ± 2.90% and 25.57 ± 2.92%), while the number of native beta sheets had increased significantly in electro-hydrogels to 15.98 ± 3.39% compared to 4.86 ± 1.90% in sonicated hydrogels. The switch in secondary structure for both hydrogels was readily apparent when they were compared to the untreated silk film control, which contained less intermolecular beta sheet and beta turn content and more amorphous structures. Substantial differences in secondary structures of sonicated hydrogels and air-dried silk films (Silk I) were supported by the low correlation coefficient (Supplementary Table S2) (0.25). In contrast, electro-gels had a higher correlation coefficient (0.60) indicating that these hydrogels were more similar to a Silk I secondary structure.

The crystallinity of the leached silk was also assessed (Fig. 1d). These results indicated that the silk released from the electro-gel had significantly more native beta sheets, and less intermolecular beta sheets and antiparallel amyloid beta sheets than the silk released from sonicated hydrogels. Both leached electro-gel samples (28.881 ± 7.08%) and leached sonicated hydrogel samples (22.125 ± 1.12%) also contained significantly less alpha and random coil structures than silk solution controls (48.00 ± 0.55%). Large standard deviations were noted for the leached electro-gel samples. However, outlier analysis produced z-scores that were not significant. Leached sonicated and leached electro-gel silk samples had a lower correlation coefficient to air dried silk than electro-gels [0.37 and 0.41, respectively (Supplementary Table S2)]. This indicated that these leached samples were less like air-dried Silk I (or the electro-gels). The correlation coefficient when compared to freeze dried silk solution indicated that electro-gels were most similar (0.98) whilst the sonicated hydrogel was least similar (0.33). Whilst the silk released from electro-gels had a high correlation to freeze dried silk (0.96), the silk released from sonicated hydrogels did not (0.42). These results indicate leached samples were not identical to the silk hydrogels nor the freeze dried silk. This observation of an ‘intermediary silk secondary structure’ was supported by thermal analysis (detailed below).

Thermogravimetric analysis (TGA) was used to determine the effect of gelation method on the thermal stability of silk hydrogels (Supplementary Table S1, Fig. S5). Silk hydrogels and leached silk contained 8–11% w/w adsorbed water. The onset of decomposition of sonicated gels (248.6 °C) was significantly delayed compared to the freeze-dried powder, silk I control (237.6 °C). Compared to the sonicated hydrogels, the electro-gels showed a reduced thermostability, but the onset of decomposition (244.1 and 245.7 °C, respectively) remained significantly higher than that of the silk I control. The onset of decomposition of leached silk obtained from electro-gels (240.7 °C) were comparable to the silk I control. This lower stability to thermal degradation suggests that the leached silk secondary structure is composed of a lower crystalline fraction compared to the hydrogel architecture and the electro-gel leached portion is similar to the silk I control.

Differential scanning calorimetry (DSC) measurements confirmed that hydrogels manufactured using sonication were composed of a greater crystalline fraction than electro-gels, and both gel types were more crystalline than the silk I control. The silk leached from both hydrogels was significantly less crystalline than the silk retained in the hydrogels, with a similar crystallinity to the silk I control. The desorption enthalpy required to remove adsorbed water ranged between − 138.4 and − 204.7 J g^−1^. Compared to the silk I control (− 235.8 J g^−1^), the desorption enthalpy was significantly lower for the hydrogel (− 163.6 J g^−1^) and leached silk (− 138.4 g^−1^) obtained by sonication. The onset of desorption ranged from 41.8 to 51.2 °C and was significantly lowered from 48.5 °C for the silk I control to 41.8 °C for the sonicated hydrogels. The glass transition of the sonicated hydrogels at 194.4 °C was also shifted to a higher temperature compared to the silk I control (182.0 °C). This suggests that the amorphous secondary structure content of silk molecules was reduced upon their integration into the hydrogel architecture, while the leached silk from both hydrogels shows a similar molecular mobility to the silk I control. The onset of the random coil to β-sheet crystallization, present for the silk I control at 207.4 °C, was delayed for the sonicated hydrogels (235.3 °C) and their leached silk (217.3 °C). Similarly, the enthalpy of crystallization for the sonicated hydrogels (2.163 J g^−1^) and electro-gels (11.60 J g^−1^) were significantly lower than the silk I control (59.44 J g^−1^). The enthalpy of crystallization of the silk leached from the sonicated hydrogels (32.02 J g^−1^) and the electro-gels (25.10 J g^−1^) were higher than those of the bulk hydrogels but remained lower than that of the silk I control. The temperature of the maximum rate of decomposition of electro-gels (267.2 °C), sonicated hydrogels (267.8 °C) and the leached portion of sonicated hydrogels (267.2 °C) were significantly higher than that of the silk I control (262.6 °C). In contrast, the temperature of maximum rate of decomposition of the silk leached from the silk leached from the electro-gels (254.8 °C) was lower than for the silk I control.

The protein size distribution in silk solution and from leached samples was examined using SDS PAGE (Fig. 2d,e). The silk solution and freeze-dried silk solution showed a longer peak from 200 to 31 kDa whereas both the sonicated and electro-gel samples showed a peak from 200 to ~ 36.5 kDa which suggested proteins > 36.5 to 6 kDa were no longer present. Freeze dried silk solution showed a band at ~ 31 kDa which can also be seen in the sonicated hydrogel but not in the electro-gel or silk solution samples. The sonicated hydrogel also had less intensity with the bands below 6 kDa than sonicated and both silk solution samples, indicating these silk fibroin fragments were present in a lower quantities.

Scanning electron microscopy (SEM) of sonicated hydrogels showed a porous network of silk with sharp edges while electro-gels appeared smoother with an interconnected structure (Fig. S6). The leached electro-gel and sonicated hydrogel samples appeared very different in their structure with a fibrous and lamellar structure, respectively. Leached silk from the electro-gel also showed an abundance of micro-sized droplet-like structures that were rare in leached silk from sonicated hydrogels.

**Figure 3:**
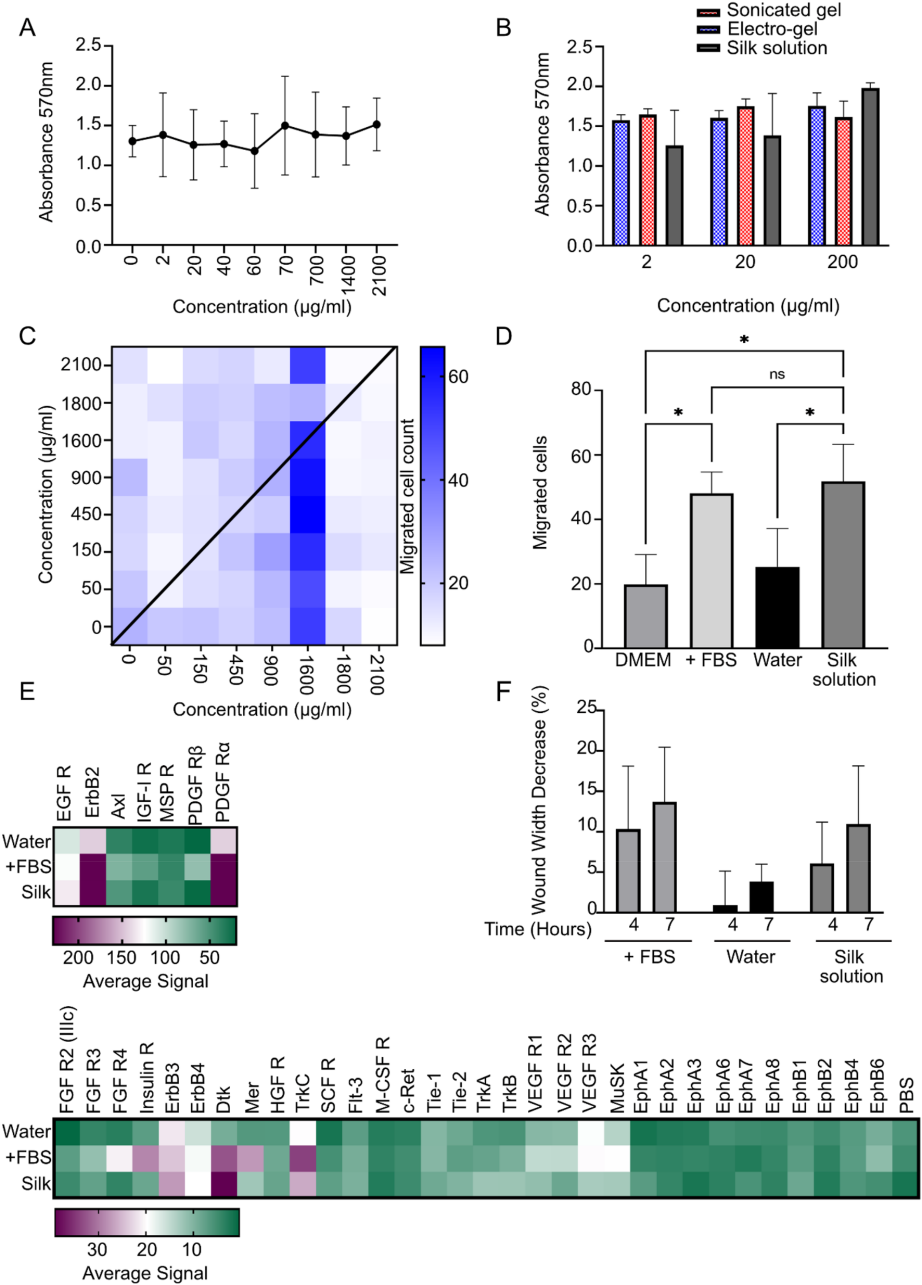
In vitro studies with silk fibroin. NIH 3T3 mouse fibroblasts were used throughout. (**A**) Cell proliferation of NIH3T3 fibroblasts incubated with silk solution for 72 h and cell viability measured with an MTT assay (n = 3). (**B**) Cell proliferation of NIH3T3 fibroblasts incubated with leached samples of silk from electro-gels, sonicated gels or silk solution after 72 h (n = 3). (**C**) Checkerboard migration assay. Cell migration across a permeable membrane after exposure to silk solution for 3.5 h (n = 4). The X and Y axes depict silk concentrations above and below the filter, respectively. Below the filter is the receiver chamber. (**D**) Cell migration at 1600 µg/mL silk solution and controls with the presence of FBS as a positive control and water as the negative control (n = 4). (**E**) Phosphorylation array in the presence of silk solution, water or FBS. (**F**) Wound closure assay width over 4 and 7 h in the presence of silk solution, water or FBS (n = 3).

The impact of leached silk on the fibroblast response was also assessed in vitro (Fig. 3, Supplementary Fig. S1). First, the influence of soluble silk on cell proliferation was determined. A broad concentration range, from 0 to 2100 µg/mL silk, was used (Fig. 3a). For these studies, the culture medium was spiked with silk. No significant effect was observed on cell proliferation over this broad concentration range. Possible differences in cellular response were taken into account by comparing the silk solution to leached silk samples collected from both sonicated and electro-hydrogels. These samples were tested at three concentrations (2, 20 and 200 μg/mL) and compared to fresh silk solution samples. No significant differences were detected among any of these samples (Fig. 3b).

Cell migration is critical for wound healing; therefore, the response of fibroblasts to soluble silk was tested using a checkerboard design to differentiate between chemokinesis and chemotaxis (Fig. 3c). The presence of soluble silk fibroin substantially influenced the movement of NIH3T3 fibroblasts. Little chemotactic movement was observed from a low to a high concentration, but NIH3T3 cells responded in particular to the 1600 µg/mL silk fibroin concentration by displaying a marked increase in cellular mobility indicative of chemokinesis. The 1600 µg/mL silk fibroin concentration was compared to a positive control (FBS) and negative control (water only) (Fig. 3d). The negative control showed a low number of migrating cells (25 ± 11.89), while FBS positive control showed a similar number of migrated cells (no statistical difference between FBS and silk fibroin).

The in vitro scratch assay was used to measure functional outcomes in response to soluble silk fibroin (Fig. 3f). Wound site reduction assessed at both 4 and 7 h showed similar trends for the different treatment groups at both time points (Supplementary Fig. S3). The presence of 1600 µg/mL silk fibroin significantly increased the rate of healing by reducing the wound site distance by 11 ± 7% at 7 h, whereas, in the absence of silk, the wound distance was reduced by only 4 ± 2%. FBS, which served as the positive control, gave a 14 ± 7% reduction in the wound site, thereby matching the silk fibroin response.

The molecular response of fibroblasts exposed to soluble silk was assessed by monitoring the tyrosine phosphorylation of receptor tyrosine kinases (Fig. 3e). The epidermal growth factor family of receptor tyrosine kinases were impacted by soluble silk fibroin. For example, ErbB2 and to a lesser extent ErbB3, were phosphorylated in response to both soluble silk fibroin and the positive FBS control. ErbB4 was also activated, albeit to a lower extent. Strong activations in response to both soluble silk and FBS were observed for PDGFα. Soluble silk induced a stronger activation of Dtk than was observed for the FBS control. FBS also simulated the receptor tyrosine kinase TrkC receptor, but this response was not observed in cells treated with soluble silk fibroin.

## Discussion

Silk fibroin has desirable material attributes^14,29^, including biocompatibility across a broad spectrum of applications (e.g. blood^30–32^, brain^33–36^, musculoskeletal^37^ etc.). The ability to unspin the silk fibre and apply diverse processing strategies to liquid silk has opened up a wide material landscape^38^. For example, silk fibroin hydrogels are being explored for numerous applications, including wound care^13^. Silk hydrogels can be generated using covalent crosslinking, physical crosslinking or a combination of both^39,40^. For this work, we used physical crosslinking by sonication energy and DC current to tune the secondary conformation. These manufacturing regimens are robust for triggering the solution–gel transition without the need for chemical crosslinkers, which can be toxic^40^. However, our current understanding of silk leaching from physically crosslinked hydrogels is limited. Obtaining a better understanding is important because preclinical assessments of silk hydrogels have overlooked silk leaching as a potential mechanism of action.

The efficiency of the solution-gel transition for electro-hydrogels is significantly lower than for sonicated hydrogels (which approaches 100% efficiency), the relative amount of silk incorporated into sonicated hydrogels was significantly lower than the amount incorporated into electro-hydrogels. Hydrogel assembly at the positive electrode is triggered by the hydrolysis of water, which creates a local drop in pH that drives hydrogel self-assembly^41^. The silk content for electro-hydrogels varied significantly, possibly due to a change in the electric field triggered by the build-up of the hydrogel at the positive electrode. We used a relatively low current, creating an electric field of 3.5 V/cm, whereas other studies have reported using a constant voltage of 25 V or an electric field of 50 V/cm^42^. We specifically selected this low voltage to determine if these conditions enable hydrogel formation because this lower voltage is more suited to the healthcare contexts (e.g. in situ hydrogel formation in the wound bed etc.). This lower voltage also created electro-gels that leached silk to ultimately trigger cell migration.

We verified the silk secondary structure of both electro-hydrogels and sonicated hydrogels. Sonicated hydrogels were rich in beta sheets, confirming the transition from silk I to silk II. By contrast, electro-hydrogels contained significantly lower amounts of beta sheets and retained the more amorphous silk I state that more closely resembled native silk or a regenerated silk fibroin solution. These data are in good agreement with the literature^43,44^, despite the use of substantially higher voltages in these earlier studies^41–43^. This suggests that a critical threshold exists for triggering silk self-assembly, but little difference occurs in the secondary structure once that threshold is passed. However, one difference may be the solid content of the hydrogel and how tightly the silk fibroin protein is packed within the hydrogel. Overall, the use of sonication and DC current enables the assembly of physically cross-linked hydrogels with tuned crystallinity that impacts both the physical and biological properties.

Based on previous work with films and scaffolds^45^, the silk crystallinity is also expected to affect the solution stability and the degradation of the silk hydrogels, but experimental proof is limited. We show here that electro-hydrogels leached significantly more silk protein over 72 h into both water and PBS when compared with sonicated hydrogels that have a high beta sheet content. When comparing the sonicated hydrogels to the electro-gels, the lower desorption temperature and enthalpy, combined with the lower enthalpy of crystallization, shallower glass transition profile and delayed onset of decomposition suggest that the sonicated hydrogels contain a greater crystalline fraction than the electro-gels (Supplementary Table S1, Fig. S5). Both hydrogel types show a higher crystallinity than the silk I control, due to the higher enthalpies of crystallization, shallower glass transitions and higher onsets of decomposition of the hydrogels compared to the silk I control. The silk secondary conformation therefore appears to be a key factor determining silk leaching because the silk is more amorphous in electro-hydrogels than in sonicated silk hydrogels. This suggests that electro-hydrogels have a less tightly bound silk structure that is prone to leaching, as nearly half the original silk content was lost over 72 h. By contrast, sonicated hydrogels released only very small amounts of silk (as a percentage in relation to the total amount present), indicating that crystallinity locks silk into the hydrogel structure and leaves little free silk that can escape. Morphological differences were apparent too. Sonicated hydrogels showed porous sharp features that were mostly absent in electro-gels. One might speculate that these sharp features were due to tighter silk binding mediated by beta sheet–rich silk fibroin structures. Morphological differences of the leached silk were evident too; the high abundance of globular structures was typical for leached electro-gel samples and are a hallmark for silk I^43^.

Unexpectedly, the silk protein released from both hydrogel types can be beta-sheet rich. This is especially surprising for the silk leached from the electro-gel. The differences in correlation coefficients for leached silk indicated that the beta sheet content was higher than pristine silk I. However, the silk secondary structure of the leached silks was also different to the respective hydrogel (based on FTIR and thermal analyses). The lower onsets of crystallization, larger crystallization exotherms, steeper glass transition profiles and lower onset of decomposition of leached silk compared to the hydrogel architecture (Table S1, Fig. S5) reinforced the greater amorphous content of leached silk determined by FTIR. This indicates that the leached silk had undergone a conformational transition forming an ‘intermediary silk secondary structure’. We speculate that this conformational switching occurred during the incubation period in the bulk aqueous phase, rather than during the integration into the hydrogel network, because (i) beta sheets provide physical anchoring points within the silk hydrogel network, thereby impeding leaching and (ii) silk in solution has a greater degree of conformation flexibility than when integrated into a hydrogel network. Overall, this work proves that silk hydrogels leach silk.

We also assessed the impact of leached silk on cell behaviour via a fibroblast model. This is an important initial assessment because the cellular response is critical for wound healing. However, the underlying mechanism by which silk fibroin hydrogels improve wound healing is currently poorly understood and is expected to be multifaceted. Other studies have shown that both in vitro and in vivo self-assembled silk hydrogels upregulated talin 1 expression, resulting in increased cell proliferation and expression of adhesion/migration related proteins^46^. Similarly, soluble silk increased the in vitro phosphorylation of ERK1/2, c-Jun and JNK1/2^47^. Soluble silk also activated NF-κB signalling both in vitro, and in a rat wound model^48^. NF-κB activation upregulated the expression of cyclin D1, vimentin, fibronectin and vascular endothelial growth factor^48^. Silk proteins have also been shown to regulate Notch signalling by suppressing *Hes-1*^49^. Other studies with silk-gelatin bioinks have shown that there is a negative regulatory role on the IHH signalling pathway and Wnt/β-catenin signalling pathway to control hypertrophy in bone marrow stromal cells^50,51^.

In the present study, fibroblasts exposed to soluble silk were assessed for the activation status of 39 key proteins. For example, the ErBb family is capable of homo-, heterodimer and higher order oligomer formation, which is orchestrated by growth factor ligands. ErbB3 showed an increased expression in silk-treated cells when compared to cells exposed only to water or supplemented medium. ErbB3 can form heterodimers with other members of the ErbB family, including ErbB2, which was also activated by exposure to soluble silk. ErbB signalling ultimately stimulates intracellular protein-tyrosine kinase activity. Autophosphorylation can also occur, and this can initiate signal transduction cascades (e.g. MAPK, Akt, JNK etc.) that regulate a plethora of cellular responses, including cell proliferation. AXL is also involved in cell proliferation and survival^52^. The activation level of AXL in response to silk was similar to that in the controls. Signalling pathways downstream of AXL include NF-κB, which was not assessed here but has been reported to be activated by soluble silk^48^. Dtk activation was higher in the silk samples than in the controls, and Dtk signalling is associated with Axl and Mer^53^. PDGFα was highly activated and affected cell signalling pathways that regulate cell growth and differentiation. The receptor tyrosine kinases TrKA and TrKB were not changed, but the expression of TrkC was lower in silk-treated cells than in the medium-treated controls. This TrkC activation could impact several other pathways, including the phosphorylation of PI3 Kinase^54^. Overall, this snapshot of receptor tyrosine kinase signalling showed that soluble silk, similar to FBS, supported fibroblast functions relevant for cell survival, proliferation and motility.

We also determined the impact of leached silk on cell function. Silk fibroin release from physically crosslinked hydrogels had little impact on fibroblast proliferation or cytotoxicity. No significant difference was detected in the biological response towards leached silk and a silk fibroin solution over the tested concentration range. These findings are at odds with the work by Park et al.^48^, who found that the presence of silk fibroin induced a dose-dependent cell growth. A possible explanation for this discrepancy could be differences in the silk processing protocols that could result in different silk molecular weight distributions.

Cell migration is a key aspect of wound healing. We therefore assessed the effects of soluble silk on cell migration and whether migration might be mediated by a gradient (i.e. chemotaxis) or by a stimulation of cell mobility (i.e. chemokinesis). The measured response indicated that silk was not chemotactic but instead induced significant chemokinesis, especially at a concentration of 1600 µg/mL. The observed migration at the key silk concentration was unexpected. We predicted cell migration to increase with concentration and to remain at a high level. We speculate that we observed off-target receptor-mediated migration. This relatively high silk concentrations triggered chemokinesis (i.e. the signalling threshold) suggesting an off-target effect rather than high fidelity receptor activation. Silk concentrations beyond this threshold abrogated migration possibly by receptor down regulation. Our experimental results suggest a complex biological response towards silk that requires more work to elucidate the mechanism of action. However, we expect that the principle of leached silk to mediated chemokinesis is relevant both in vitro and in vivo. Gradient formation and high local silk concentration are feasible and can be further tuned by adjusting the volume of administered hydrogel. The in vitro scratch wound assay showed that the presence of soluble silk fibroin caused the cells to migrate more rapidly into the scratch than they did in the absence of silk. Cell migration is dependent upon microtubule polymerization of the cytoskeleton. The cellular mechanism of action of silk is currently not fully known, but one possibility is that the VITTDSDGNE and NINDFDED sequences found in the N-terminal region of silk fibroin^55^ promoted chemokinesis in fibroblasts. Overall, silk is emerging as a powerful fibroblast chemokinesis stimulator, suggesting that the presence of leached silk could accelerate this aspect of wound healing. This hypothesis is supported by in vivo data in a corneal wound model where animals treated with liquid silk showed significantly faster wound healing than saline controls^27^. Similar observations were also made for a skin wound model in rats^48^.

## Conclusion

Silk hydrogels with tuned secondary structures can be formed by sonication or electro-gelation. The elution of silk from the hydrogels is significantly greater for electro-hydrogels, due to their more amorphous structure and lower beta sheet secondary structure. The leached silk from such gels is biologically active. No significant effect was noted on cell proliferation; however, soluble silk promoted the phosphorylation of receptor tyrosine kinases and stimulated chemokinesis. These effects are desirable for wound management; therefore, silk fibroin hydrogels show promise for wide use in the healthcare setting.

## Materials and methods

### Silk solution and hydrogel manufacture

The silk solution was prepared as detailed previously^56,57^ and described in video format elsewhere^58^. Briefly, *Bombyx mori* silk cocoons were cut into 5 × 5 mm pieces and 5 g samples were degummed in 0.02 M Na_2_CO_3_ solution for 30 min. The purified silk fibroin was given three 20 min rinses in Milli-Q ultrapure water and then stretched and left to dry overnight. The dried silk fibroin fibres were then packed into a beaker and dissolved in a 9.3 M LiBr solution at 60 °C for up to 4 h. The resulting solution was dialyzed (MW cut off 3500 g/mol, Thermo Scientific, Waltham, MA) for 48 h in Milli-Q water to remove the LiBr salt. The resulting silk fibroin solution was cleared by centrifuging twice at 9418×*g* and 5 °C for 20 min. The silk fibroin content was determined gravimetrically. The silk fibroin solution was stored at 4 °C until use.

Physically cross-linked silk fibroin hydrogels were manufactured using a 5% w/v silk fibroin solution stock and exposing this solution to either sonication or electro-gelation. For the former, the silk hydrogels were prepared as detailed previously^36^. Briefly, a digitally controlled probe sonicator (Sonoplus HD 2070, Bandelin, Berlin, Germany) fitted with a 23 cm long sonication tip (0.3 cm diameter tip and tapered over 8 cm) was used. Unless otherwise stated, 5 mL sample batches in 15 mL Falcon tubes (1.4 cm diameter and 11 cm long) (Greiner Bio-One GmbH, Kremsmünster, Austria) were exposed on ice to a 45% amplitude for typically 3 to 6 sonication cycles (one cycle consisted of 30 s on and 30 s off) to induce the solution-gel transition. Electro-gelation of the silk solution was performed by adding 10 mL of silk fibroin to a glass beaker (4 cm diameter and 3.5 cm tall) and stainless-steel electrodes (1 × 2.5 × 0.1 cm) were submerged to a depth of 1 cm. The electrodes were connected to a Solartron SI 1286 instrument (Ametek, Hampshire, UK) and a galvanostatic input was set to 0.5 mA for 90 min. The resulting gel was removed from the positive electrode and used immediately.

The quantity of silk incorporated into each silk fibroin hydrogel was determined by gravimetric analysis. The silk hydrogel production efficiency was calculated by taking into account the solid content of the hydrogel and comparing to the amount of silk in the starting solution.

The secondary structures of silk samples were determined by Fourier transform infrared (FTIR) spectroscopy (Tensor II Bench ATR IR, Bruker, MA, USA). Each FTIR measurement was run for 128 scans at a 4 cm^−1^ resolution in absorption mode over the wavenumber range of 400–4000 cm^−1^. Silk hydrogel samples were dried before undergoing FTIR analysis. The silk solution after the electro-gelation was assessed by collecting the solution and drying the samples prior to FTIR analysis. For silk leaching experiments (detailed below), the samples were freeze-dried (Epsilon 2-4, Martin Christ, Germany). Reference samples with a low and high beta sheet content were prepared by drying the silk fibroin solution to form a film. A high beta sheet (silk II) content was induced by treating the film with 70% v/v ethanol for 2 h (silk II), whilst low beta sheet films were left untreated (silk I). All FTIR data were deconvoluted as described previously^59^. The correlation coefficient was calculated using air-dried silk film or freeze-dried silk solution as the comparator for all samples while the second derivative of the absorbance spectra was processed and smoothed with a seven-point Savitzky–Golay function with a polynomial order of 2 as detailed previously^56^. Outlier analysis was performed where high variance was observed with Z-scores. Significance (Z > 2).

### Silk leaching from hydrogels

Silk leaching from each hydrogel was quantified using a micro bicinchoninic acid (BCA) protein assay (Pierce Biotechnology, Thermo Scientific, IL, USA). Briefly, a known hydrogel sample was placed into a 1.5 mL Eppendorf tube and either ultrapure water or phosphate buffered saline (PBS, Sigma Aldrich, MO, USA) was added at a ratio of 1 mL per 0.02 g of wet hydrogel mass. Static leaching experiments were performed at 37 °C. At the desired time point, the supernatant was quantitatively removed and replaced with fresh solution. The collected samples were stored at 4 °C for up to 48 h. The BCA assay was performed according to the manufacturer’s instructions, except that a silk fibroin standard curve was used for quantification. Unknown samples were interpolated using GraphPad Prism software (GraphPad Software Inc, CA, USA).

The collected samples were also subjected to sodium dodecyl sulphate polyacrylamide gel electrophoresis (SDS-PAGE) to assess the fibroin molecular weights. Samples were lyophilized and reconstituted in Milli-Q water at a concentration of 5 mg/mL^60^. The protein concentration was determined with the BCA assay, and the silk samples were diluted in Milli-Q water to 5 mg/mL. All samples were separated using a mini-PROTEAN TGX Precast Gel (4–20%) (Bio-Rad, Hertfordshire, UK) and a running buffer consisting of 25 mM Tris, 192 mM glycine and 0.1% SDS, pH 8.3. Samples were reduced using 2 × Laemmli buffer and β-mercaptoethanol, followed by heating at 90 °C for 5 min. A 10 µL sample was then loaded and separated at 200 V, with a 30–35 mA current for 30–40 min. The gel was stained with the SilverXpress Staining Kit according to the manufacturer’s instructions (ThermoFisher, Renfrew, UK). Images were acquired with a 12.2 MP megapixel Google Pixel 3 phone, and the pixel density was analysed by ImageJ v1.52n (National Institutes of Health, Bethesda, MD, USA).

### Thermal analysis of silk hydrogels and leached silk

A known volume and mass of each silk sample and freeze-dried silk control was frozen at − 20 °C overnight, followed by freeze-drying for 20 h at − 10 °C and 0.14 mbar. First-cycle differential scanning calorimetry and thermogravimetric analyses were carried out on the dried samples (2.02–2.34 mg) in aluminum pans from 25 to 270 °C at a scanning rate of 10 °C min^−1^ and under a nitrogen flow of 20 mL min^−1^ (TGA 2 and DSC 3+, Mettler-Toledo Ltd., OH, USA). The mass of each sample used was equivalent for thermogravimetry and differential scanning calorimetry. Thermograms were analyzed using STARe Evaluation Software v16.3 (Mettler-Toledo Ltd., OH, USA). The glass transition was characterized according to ASTM E1356, and the glass transition and change in heat capacity are reported as the ISO midpoint and the ISO ΔC_p_. DTA and DDSC were calculated in STARe Evaluation Software v16.3 as the first differential of the TGA and DSC curves, respectively, using a 13-point smoothing function.

### Scanning electron microscopy

To image the hydrogels and leached samples, known quantities of each were freeze-dried before samples were attached to aluminium stubs with carbon adhesive pads. Samples were sputter coated (ACE200, Leica Microsystems, Wetzlar, Germany) with gold to reduce charging in the SEM. Samples were viewed with a Hitachi SU6600 Field Emission SEM at voltage 5 kV under standard vacuum settings at magnifications of 300 ×, 1000 × and 10,000 ×.

### Cell response towards silk

NIH-3T3 cells (ATCC CRL-1658, ATCC, England, UK) were cultured in Dulbecco’s Modified Eagle’s Medium (DMEM, ThermoFisher Scientific, Paisley, UK) supplemented with 10% v/v foetal bovine serum, 50 U/mL penicillin and 50 µg/mL streptomycin. Culture conditions were maintained within a humid incubator set at 37 °C and 5% CO_2_.

The impact soluble silk on cell proliferation was assessed by seeding NIH-3T3 cells into 96-well tissue culture treated polystyrene plates (Corning Inc., Costar, Kennebunk ME, USA) at 5 × 10^4^ cells/cm^2^ in 100 µL of culture medium. The plates were then incubated for 24 h before the medium was removed. A total of 18 wells across 3 repeats were used. A gradient of silk solution (volume 30 µL) ranging from 0 µg/mL to 2100 µg/mL in culture medium, was then added to each well. After 72 h, an MTT assay was performed by adding 20 µL (3-(4,5-dimethylthiazol-2-yl)-2,5-diphenyltetrazolium bromide (MTT) (5 mg/mL in PBS) and incubating for 5 h. The MTT was then aspirated and 100 µL dimethyl sulphoxide was added to dissolve the formazan crystals. After a further 15 min incubation, the absorbance at 570 nm was read on a spectrophotometer (Thermo Multiskan Ascent, Thermo Labsystems, Finland).

For migration studies, NIH-3T3 cells were washed with PBS and starved in un-supplemented DMEM overnight. The cells were then harvested with Accutase cell detachment solution (BioLegend, San Diego CA, USA), stopped with soyabean extract and resuspended in non-supplemented DMEM. ChemoTx 96 well disposable chemotaxis plates (Neuroprobe, Gaithersburg MD, USA; Supplementary Fig. S2) were coated with 1 µg/mL fibronectin (Sigma Aldrich, Scotland, UK) and a soluble silk gradient (0–1600 µg/mL contributing 10% v/v in unsupplemented DMEM) and controls (medium with 10% v/v FBS or 10% v/v water) were pipetted into the receiver wells. The filter was replaced, and contact between the surface of the liquid and cell site area was ensured. These checkerboard assays also used a silk concentration gradient, added to the top filter, followed by 1 × 10^5^ cells/cm^2^. Samples were incubated for 3.5 h, then the cells on the underside of the filter were fixed, stained with 0.5% crystal violet (Sigma Aldrich, Scotland, UK) and counted under a microscope.

The in vitro scratch wound healing assays were performed by seeding cells into 12-well plates at a density of 5 × 10^4^ cells/cm^2^ and leaving them to attach for 24 h. The culture medium was then removed, the cells were washed with PBS, non-supplemented DMEM was added and the cells were incubated overnight. A scratch was then made with a pipette tip and the medium was removed and replaced with unsupplemented DMEM containing either water controls, 10% v/v FBS or 1600 µg/mL silk solution (final concentration). For control cultures, 1 µg/mL nocodazole (Sigma Aldrich, Paisley, Scotland) was added. The cells were imaged over 8 h (EVOS FL Auto, Thermo Fisher Scientific, California, CA, USA).

The mouse phosphorylation array (Mouse Phospho-RTK Array ARY014, R&D Systems LTD, Minneapolis, MN, USA) was used as detailed previously^61^ to examine the molecular response of fibroblasts. Briefly, cells were grown to confluence and exposed for 30 min to (i) 1600 µg/mL silk solution, (ii) water or (iii) DMEM with 10% foetal bovine serum and (iv) without any treatment. Cell lysates were then harvested on ice with lysis buffer containing 10 µg/mL aprotinin (Sigma Aldrich, Paisley, UK), 10 µg/mL leupeptin (Tocris Bioscience, Bristol, UK) and 10 µg/mL pepstatin (Tocris Bioscience). The protein content of each lysate was measured with the BCA assay. The cell lysates were then added at 200 µg protein per array and developed using autoradiography film (UltraCruz, Dallas, TX, USA). The film was digitized using a scanner (HP Envy 4250, HP Inc UK Limited, UK) and processed using Image J software v1.53c (National Institutes of Health, USA).

### Statistical analyses

Statistical analyses were carried out using Origin Pro 2018 (Northampton, Massachusetts, USA) and GraphPad Prism v.9.1.1 (San Diego, CA, USA). One-way analysis of variance, followed by Bonferroni’s post hoc test, was conducted between multiple groups, or two-way analysis of variance followed by Tukey’s multiple comparisons. Normality and homogeneity of variances were assumed. Asterisks denote statistical significance as follows: *P < 0.05, **P < 0.01, and ***P < 0.001. All data are presented as mean values ± standard deviation, and the number of independent experiments (n) is noted in each figure legend.

## Supplementary Information

Supplementary Information.

## Data Availability

All data created during this research are openly available from the University of Strathclyde-Pure, 10.15129/5bcd43c9-3af4-4097-ad9d-6acff7560572.
