# Supplementary material for: Impact of silk hydrogel secondary structure on hydrogel formation, silk leaching and in vitro response
Source: Sci Rep. 2022 Mar 8;12:3729. doi: 10.1038/s41598-022-07437-4 (PMC8904773; doi:10.1038/s41598-022-07437-4)
Supplement: Supplementary file 1 — Supplementary Information. [file 41598_2022_7437_MOESM1_ESM.pdf]

Impact of Silk Hydrogel Secondary Structure on Hydrogel Formation, Silk Leaching and In Vitro Response

Supplementary Information

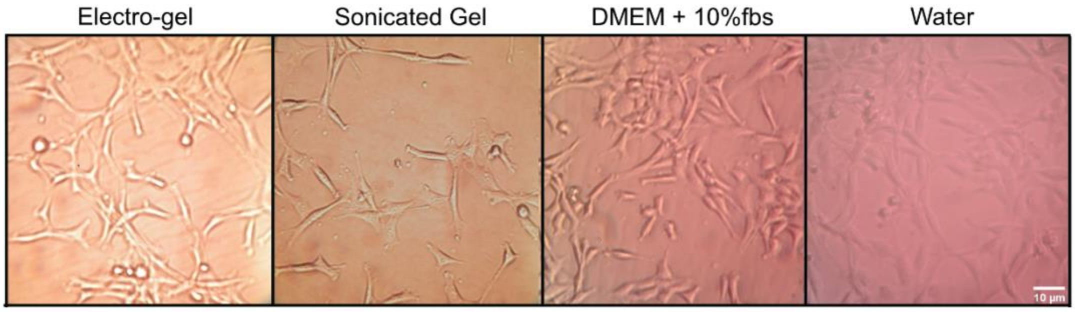

Figure S1. Cell proliferation images with 200 µg/ml of eluted silk from electro-gel and sonicated gel, 10 % fbs or water after 24 hours. Scale bar 10 µm.

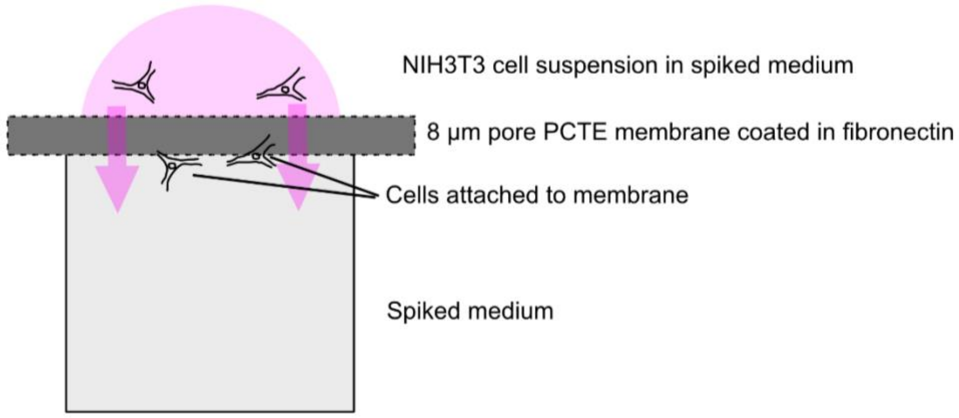

Figure S2. Cell migration assay diagram. ChemoTx disposable chemotaxis plates consisting of a PCTE membrane over a 96 well plate. Image displays the placement of spiked medium and cells. Cells can travel through the pores and will adhere to the fibronectin coated membrane for counting.

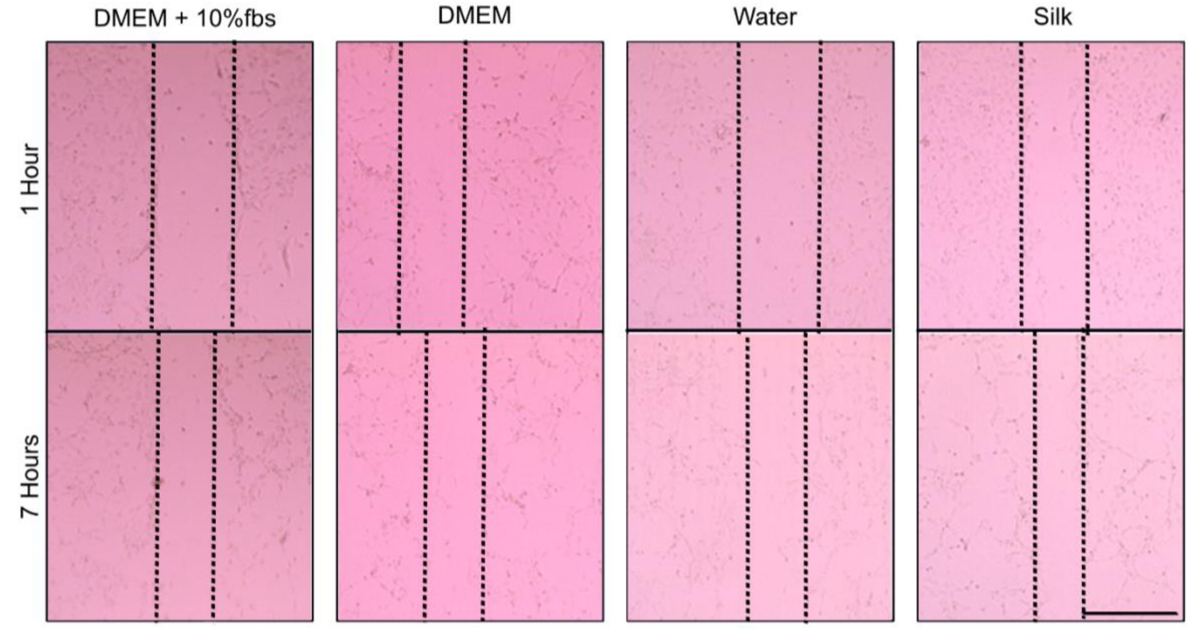

Figure S3. Wound closure assay with NIH3T3 fibroblasts at 1 and 7 hours. Broken lines indicate the border of cell growth. Scale bar 400 µm.

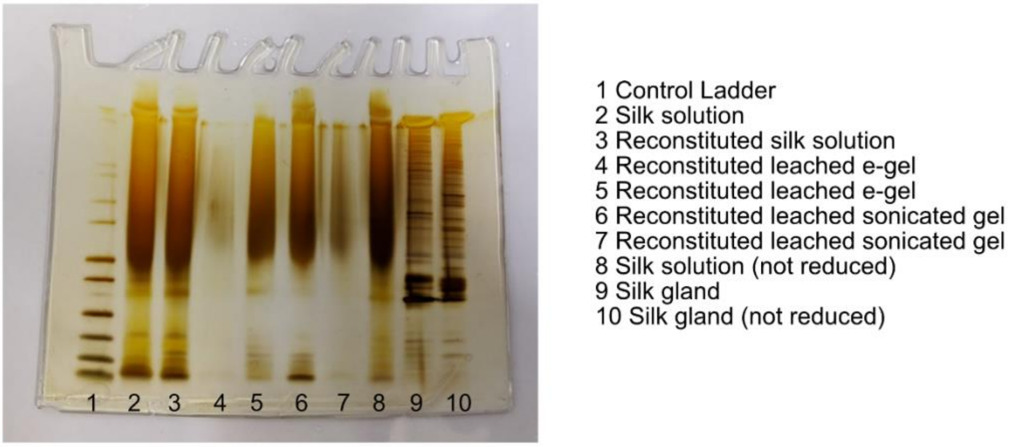

Figure S4. SDS PAGE of protein released from electro-gels and sonicated gels. Standards included are silk solution and freeze-dried silk solution reconstituted in water.

Table S1. First-cycle thermal analysis data of the gel and leached portions from sonicated gels and electro-gels.

	Thermal Property	+Silk I	Gel portion		Leached portion	
		Freeze-dried silk	Sonicated	E-gel	Sonicated	E-gel
DSC	$T_{o,des}$ / °C	48.46 ± 0.72	41.82 ± 3.40	51.04 ± 0.28	50.82 ± 0.48	50.87 ± 1.89
	T_{des} / °C	77.55 ± 2.18	75.43 ± 0.79	79.8 ± 5.56	79.52 ± 1.73	83.83 ± 2.11
	T_{des}' / °C	60.08 ± 3.27	58.12 ± 2.62	66.08 ± 1.00	64.04 ± 1.52	66.89 ± 1.97
	ΔH_{des} / J g^{-1}	-235.8 ± 23.7	-163.6 ± 27.1	-204.7 ± 15.8	-138.4 ± [illegible]	-208.4 ± 38.9
	T_g / °C	182.0 ± 1.1	194.4 ± 1.7	181.1± 0.8	187.2 ± 2.5	183.0 ± 0.7
	$T_{o,c}$ / °C	207.5 ± 1.2	235.3 ± 2.7	213.4 ± 0.8	217.3 ± 0.4	204.4 ± 5.1
	T_c / °C	250.0 ± 0.9	246.3 ± 4.9	233.8 ± 0.5	253.2 ±0.6	243.6 ± 0.4
	ΔH_c / J g^{-1}	59.44 ± 2.60	2.163 ± 2.914	11.60 ± 1.18	32.02 ± 4.57	25.10 ± 2.53
	$T_{o,dec}$ / °C	261.8 ± 0.0	263.8 ± 0.7	253.3 ± 4.5	263.0 ± 0.4	252.6 ± 0.9
	T_{dec}' / °C	262.6 ± 0.5	267.8 ± 0.8	267.2 ± 0.1	267.2 ± 0.0	254.8 ± 1.1
TGA	[H_2O] / %	11.10 ± 1.26	10.14 ± 0.30	10.05 ± 0.27	8.22 ± 0.79	11.07 ± 1.97
	$T_{o,dec}$ / °C	237.6 ± 2.8	248.6 ± 0.5	244.1 ± 1.5	245.7 ± 2.7	240.7 ± 0.7
	T_{des}' / °C	72.43 ± 5.99	66.72 ± 4.63	75.10 ± 4.83	69.78 ± 4.79	70.83 ± 1.21

Table S2. Correlation coefficient for FTIR deconvolution against air dried silk films and freeze-dried silk

Correlation Coefficient (± SD)

	Silk Film	Freeze dried silk solution
Sonicated gel	0.251550 ± 0.026454	0.332781 ± 0.092321
Electro-gel	0.602864 ± 0.136176	0.981086 ± 0.010196
Leached sonicated gel	0.368774 ± 0.045787	0.421985 ± 0.121619
Leached electro-gel	0.412926 ± 0.039905	0.962778 ± 0.019018

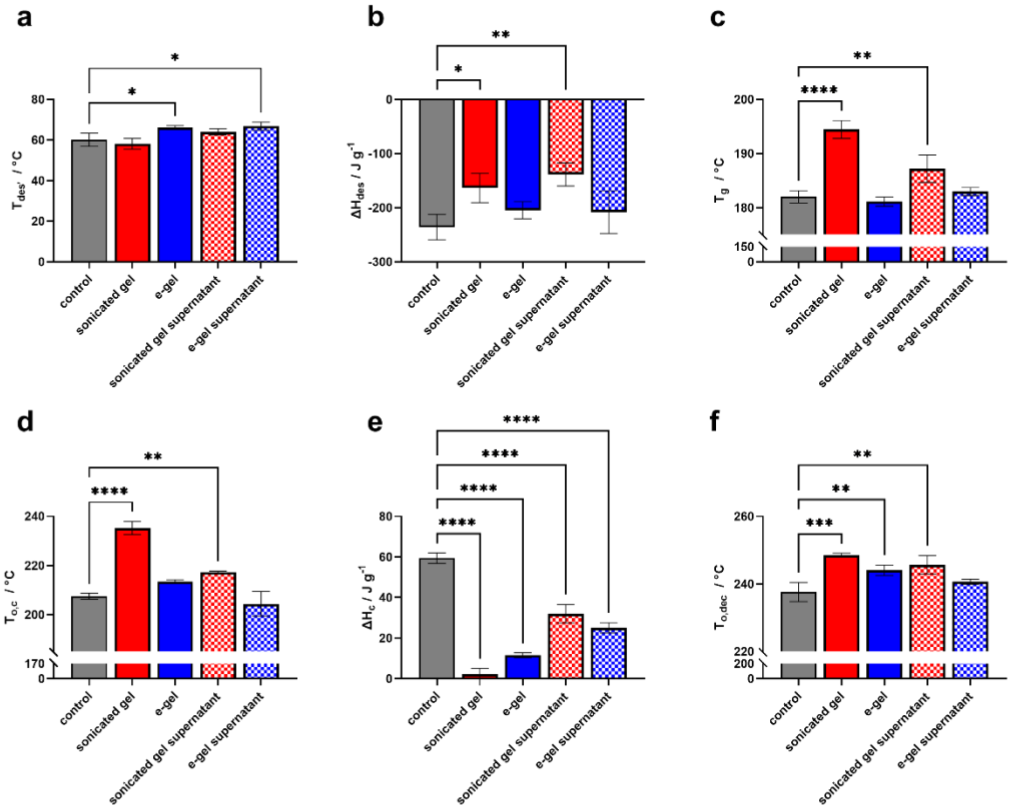

Figure S5. Sonicated silk hydrogels and the leached sonicated silk were more crystalline than electro-gels which displayed a thermal signature of intermediate crystallinity. The leached silk portion from both electro-gels and sonicated gels showed reduced crystallinity compared to the gel portion. The variation in (a) temperature of maximum rate of desorption (Tdes') (b) enthalpy of desorption (ΔHdes), (c) ISO glass transition temperature (Tg), (d) onset of crystallization (To,c), and (e) enthalpy of cold crystallization (ΔHc) from first-cycle DSC and (f) the extrapolated onset of decomposition from first-cycle TGA. Error bars are hidden in the bars and plot symbols when not visible, ± SD, n = 3. Multiple factors were evaluated by one-way analysis of variance (ANOVA), followed by Dunnett's multiple comparison post-hoc test against the control group. Asterisks denote statistical significance determined using post-hoc tests as follows: *p < 0.05, **p < 0.01, ***p < 0.001, ****p < 0.0001.

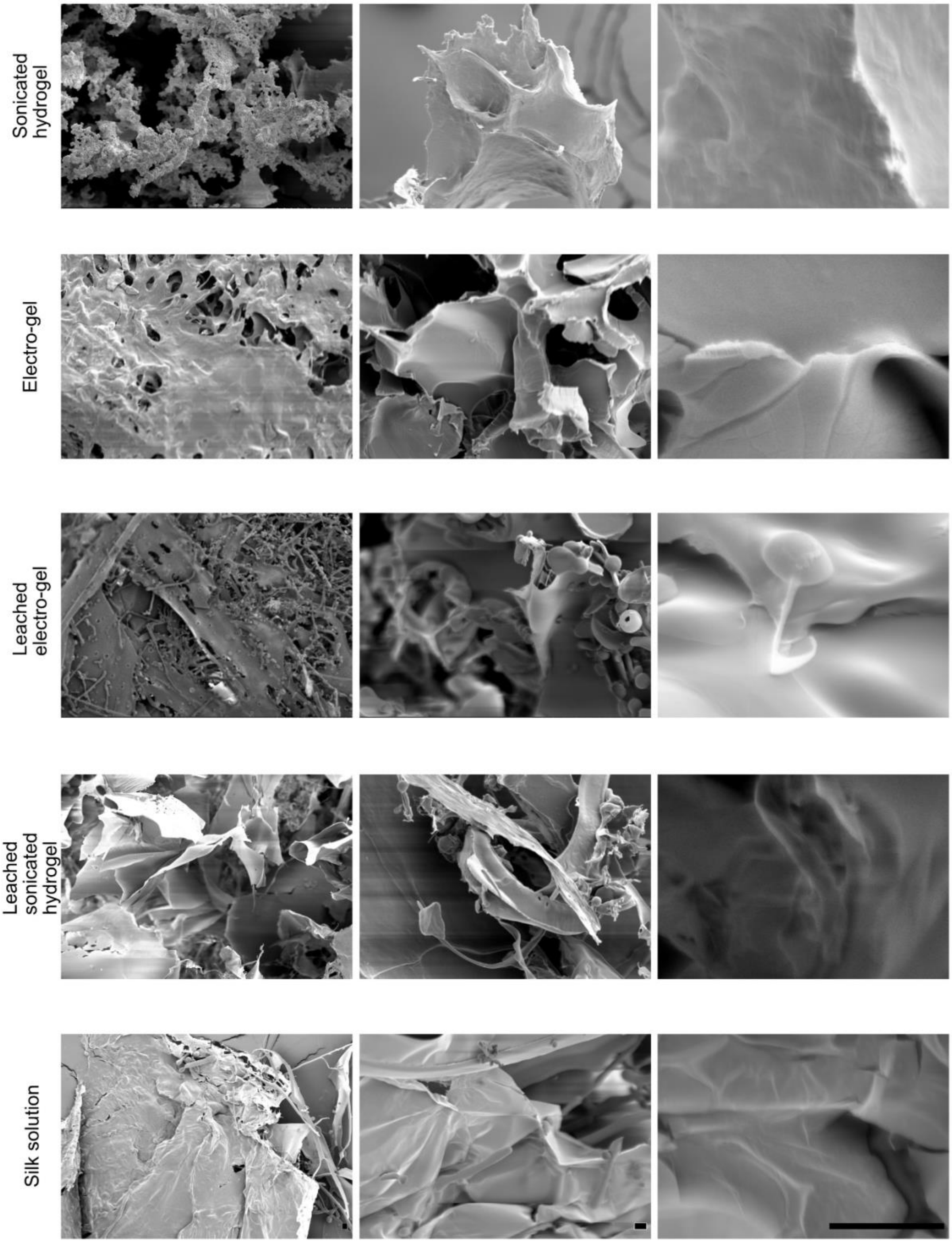

Figure S6. SEM images of sonicated hydrogels, electro-gels, and the leached silk from both. Silk solution is included as a reference. Scale bar 5 µm.

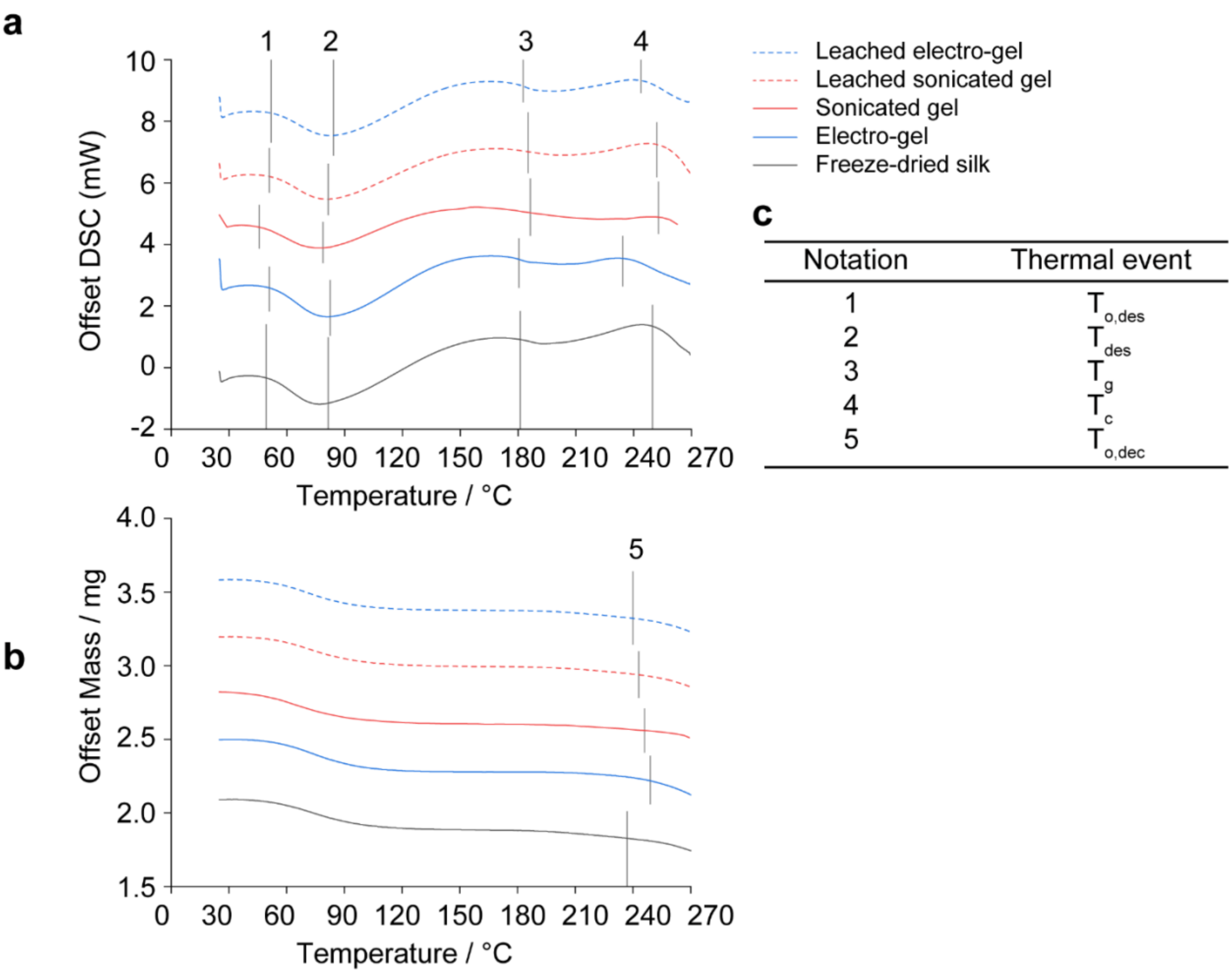

c

Notation	Thermal event
1	$T_{o,des}$
2	T_{des}
3	T_g
4	T_c
5	$T_{o,dec}$

Figure S7. Exemplary first-cycle (a) DSC and (b) TGA thermograms of sonicated gels, electro-gels and leached portions. (c) Key for thermal events specifying the onset of desorption ($T_{o,des}$), extrapolated temperature of desorption (T_{des}), ISO glass transition temperature (T_g), extrapolated temperature of crystallization (T_c) from first-cycle DSC and the onset of decomposition ($T_{o,dec}$) from first-cycle TGA.
